# Reforming schools into health promoting schools: perspective based on expert consensus from a European multistakeholder consultation

**DOI:** 10.1007/s00431-025-06736-y

**Published:** 2026-02-09

**Authors:** Konstantinos C. Makris, Christiana Philippou, Constantina Vasileiou, Michael Tornaritis, Stella Canna Michaelidou, Charalambos Hadjigeorgiou, Marina Kyriacou, Matthaios Santamouris, Itamar Grotto, Stephan Bose-O’Reilly, Joao Breda, Peter van den Hazel

**Affiliations:** 1 Cyprus International Institute for Environmental and Public Health, School of Health Sciences, Cyprus University of Technology, Limassol, Cyprus; 2 Department of Rehabilitation Sciences, School of Health Sciences, Cyprus University of Technology, Limassol, Cyprus; 3 Cyprus Ministry of Education, Sports and Youth, Nicosia, Cyprus; 4 WHO Athens Quality of Care and Patient Safety Office, World Health Organization, Regional Office for Europe, Athens, Greece; 5 Educational Institute of Child Health, Nicosia, Cyprus; 6 Cyprus National Committee for Environment and Child Health, Nicosia, Cyprus; 7 School Built Environment, Faculty Art Design and Architecture, University New South Wales, Sydney, Australia; 8 Department of Epidemiology, Biostatistics and Community Health Sciences, School of Public Health, Faculty of Health Sciences, Ben-Gurion University of the Negev, Beersheba, Israel; 9 Institute and Clinic for Occupational, Social and Environmental Medicine, University Hospital, LMU Munich, Munich, Germany; 10 International Network on Children’s Health, Environment & Safety (INCHES), Ellecom, The Netherlands

**Keywords:** Children, Schools, Wellbeing, Environmental health, Health services, Exposome

## Abstract

Every school on this planet should aim to become a healthy school. This is the motto of the WHO/UNICEF health promoting school strategic agenda (HPS). To address gaps and needs of children’s health and educational inequalities in schools, a qualitative study was set up to synthesize experts’ views and insights on school health programs and the HPS strategy in Europe. This multi-stakeholder consultation took place during an international children’s health workshop in Cyprus (October 2024). Data was collected through focus group interviews with ~ 30 experts engaged in a multistakeholder consultation between academics, governmental officials, teacher liaisons, and health professionals, including WHO experts and NGOs. The consultation identified the following themes: systemic limitations in funding and sustainability of health education/promotion programs, enhanced curriculum needs for health literacy, limitations in school physical environment, rise in behavioral risk factors among children and adolescents, and growing mental health needs. A consensus was reached regarding the prerequisites and recommendations towards improving both health and educational outcomes for children; the implementation of holistic methodological frameworks, such as that of the human exposome and its exposomics tools were collectively proposed to better deploy the HPS recommendations in the school community.

*Conclusion*: By adopting a holistic, multi-sectoral approach for schools and their communities (parents, teachers, authorities, the public) that embeds the exposomics tools and methodologies, future societies would ensure that educational institutions not only serve as centers for academic learning, but also as environments that nurture the physical, mental, and social wellbeing of students.

**Table:** 

**What is Known:** • *The importance of schools and their communities, as settings to efficiently implement health education and promotion programs for children (4–19 years of age).*	
**What is New:** • *The utility of the human exposome in integrating multiple children’s risk factors and health services that overall impact growth and development.* • *Observatory infrastructures in schools would allow for the systematic monitoring and integration of children’s health and of their multiple non-genetic risk factors, feeding into the health promoting school strategy.*

**Supplementary Information:**

The online version contains supplementary material available at 10.1007/s00431-025-06736-y.

## Introduction

Healthy children tend to learn better, and higher educational levels predict better health outcomes in adulthood [1, 2]. Children’s health is shaped by a range of factors, including physical and social environments in the settings where they live, learn, and play; good acoustic indoor quality, green school environments, adequate shade, light, and aeration are major pillars of an adequate school environment and infrastructure [3]. As these impact children’s well-being and development, schools play a crucial role in shaping these environments to be safe and supportive. The health promoting school (HPS) concept of the World Health Organization (WHO) and UNESCO is a prime example of a strategic approach to guide reforms and updates in the education system to transform all schools into healthy schools, leaving no child behind regardless of their ethnic background, socioeconomic position, etc. [4].

Such holistic and cross-sectoral approaches may be supported by novel methodological frameworks and supportive tools, like that of the human exposome and its exposomics tools [5–11]. The human exposome methodological framework was originally coined by Dr. Wild in 2005 [6] and it has been defined as the comprehensive characterization of all environmental exposures from conception till death, including its endogenous response [10]. The often-fragmented approaches for school programs are focused on a single health risk or environmental factor and they might suffer from limited effectiveness, and/or being concentrated in a conventional school sample with no longitudinal follow-up, or without adopting an inclusive for all approach; yet, it is often the case that social determinants of health are not incorporated in such programs. The human exposome concept offers tools to integrate multiple exposures with individual-level data on location (e.g., school and residential addresses or zip code), environmental, lifestyle/behavioral, or psychosocial variables together with sociodemographic data to neighborhood- and community-level data as they evolve over time [7, 8].

The human exposome concept has been already tested in children’s populations to better understand effects of multiple exposures on key children’s growth and development indices, like lung function or child behavior or blood pressure [12–14]. The U.S. Children’s Health CHEAR exposomic infrastructure allowed for pediatric research to measure a wide range of chemical exposures and health outcomes using exposomics tools [15]. The Japan Environment and Children’s Study (JECS) represents Japan’s strategy in incorporating exposomics into children’s health research [16]. The EU has invested > 100 million euros in exposome science with some of them being focused on children’s exposome, such as the EU EQUAL-LIFE exposome project on children’s mental health and cognitive development, and the EU ATHLETE exposome project addressing the health effects of multiple environmental hazards and their mixtures, starting from the earliest stages of life.

The efficient implementation of an HPS strategy in schools would greatly benefit from the application of holistic approaches. For example, the EU Schools4Health project has created an online hub dedicated to whole-school approaches to health and wellbeing, building on the HPS strategy. However, there appears to be a gap in systematically advancing and scaling up the pragmatic assessment of multiple risk factors and actionable solutions that reduce risk of exposure to such risk factors into the strategic implementation plan of HPS in school settings and their curricula.

To address such gaps and needs of children’s health and educational inequalities in schools, the study objective was to synthesize and integrate experts’ views on school health programs and the HPS strategy in Europe. This multi-stakeholder consultation took place during an international children’s health workshop in Cyprus.

## Methodology

The consultation exercise was designed as an exploratory stakeholder engagement targeting individuals with relevant expertise (“those who have the information”), in compliance with the principles and methodological guidance of the European Commission’s Better Regulation Toolbox [17]. Experts were those technically qualified, selected by their organization and positively responded to our initial invitation to participate in this consultation. Thus, the consultation strategy combined the targeted (experts) consultation method with the tools of an expert meeting and focus group interviews (guide as SI text in Appendix). Focus groups were chosen here because they represent a cost-effective and reliable mode of acquiring richer and socially interacting qualitative data. The stakeholder consultation aimed to provide broad and high-quality information to support the reform of our schools to become health promoting schools, accounting for the views of all key stakeholders. The consultation was announced as part of the 10th anniversary of the Cyprus International Public Health Workshop, which was organized in Nicosia, Cyprus, in October, 2024. The pool of potential stakeholders-experts that was invited to participate in the consultation was defined by the EU-wide network of the workshop’s committees and that of the International Network for Children’s Health, Environment and Safety (INCHES). Because of lack of funds, most experts who eventually attended the event covered stakeholder groups from the Republic of Cyprus, albeit expert participation from other European and third countries (Greece, Switzerland, the Netherlands, Germany, Slovenia, Slovakia, Australia, Israel) and stakeholder groups (international health policy organizations, medical practitioners, NGOs, academia, research organizations) was evident. Stakeholder representation was balanced across the three focus groups. The study did not include any personal data information, so ethical approval was not required. All participants provided informed consent to participate in the focus group, and the research was conducted according to the ethical principles of the Declaration of Helsinki.

The consultation approach entailed a mix of open-ended questions plus a more generic request for views on pre-selected topics in the field of children’s health and school health systems. The thematic analysis consisted of an initial process of data understanding in the transcripts, followed by code generation, grouping of codes into themes, review and revisions of the themes, naming of themes and report writing. A rapporteur of each focus group did the manual coding, followed by an iterative process within and between the focus groups for refining all generated themes, until a consensus was reached. Non-verbatim transcription was used, and the duration of focus groups was ~ 3 h. The consensus-building process proceeded as follows. Each of the three focus groups reported on existing school health policies and programs and identified key challenges currently faced by primary and secondary schools in Europe. The focus group discussions were transcribed in English by the respective leads and rapporteurs. These transcripts were then thematically coded using an inductive approach to generate the main themes and subthemes. To ensure reliability, the identified themes were independently cross-verified and agreed upon by the leads and rapporteurs from the other two focus groups. Consensus on the final themes and the corresponding set of proposed interventions and recommendations was achieved through an iterative, hierarchically inductive process. This iterative validation continued until unanimous agreement was reached among all participating experts regarding the accuracy and comprehensiveness of the synthesized themes and recommendations. This report applied the Standards for Reporting Qualitative Research (SRQR) reporting guidelines [18].

## Results

**Fig. 1:**
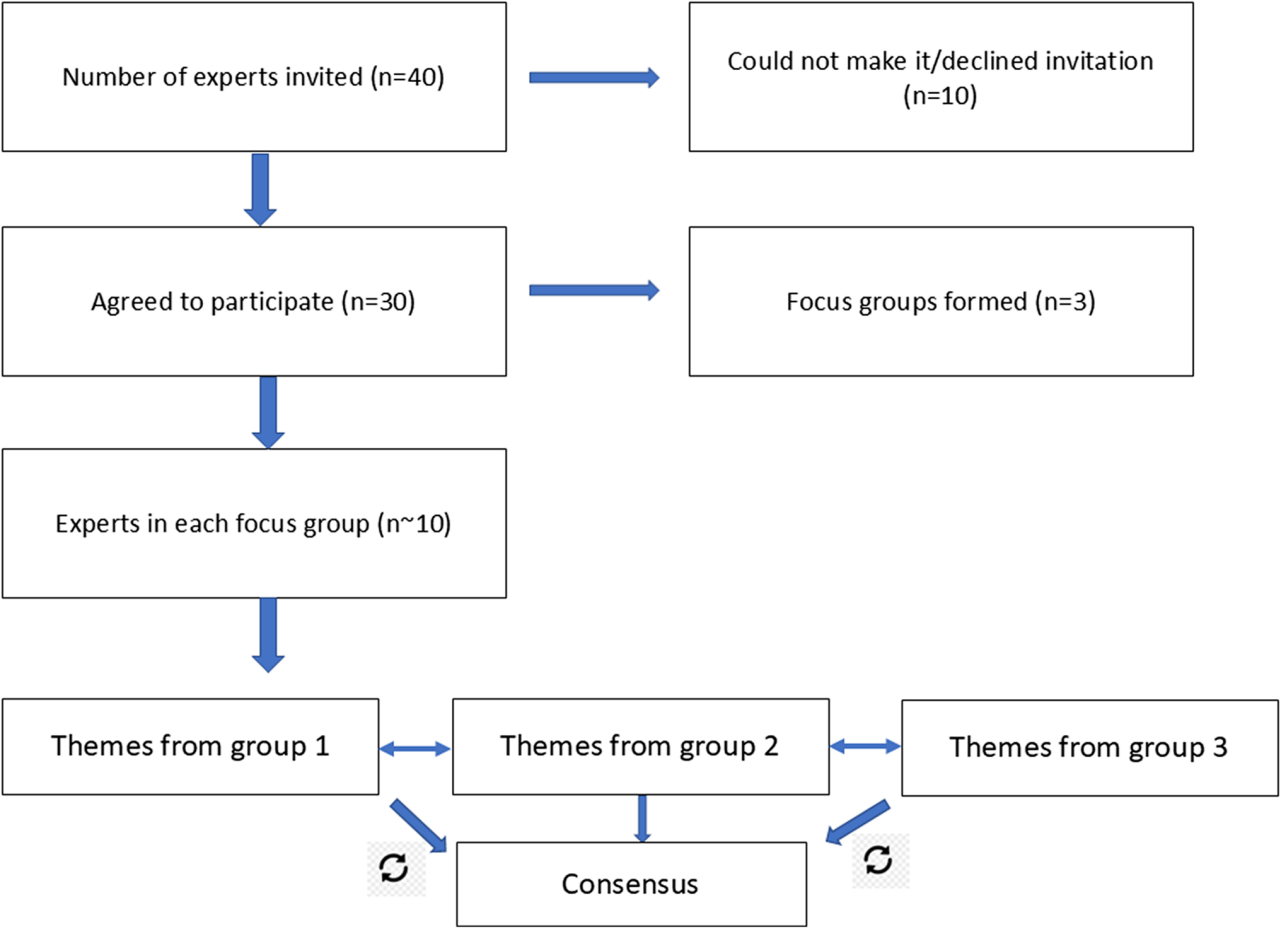
Flowchart of expert participants and the consultation approach of analyzing the focus groups’ content and themes in a loop process to reach consensus

Representative experts from the following key stakeholder groups were invited and actually participated in the consultation: public authorities and Ministries of Education, Sports and Youth and Ministry of Health (*n* = 3); citizens and parents of school age children (*n* = 2); industry, such as, wearable or stationary sensors and related health technologies (*n* = 3); private health insurance representative (*n* = 1); medical doctors and nurses communities (*n* = 2); school teachers (*n* = 7); relevant international organizations, such as experts from the World Health Organization (WHO) Headquarters (*n*=1), WHO Athens Quality of Care and Patient Safety Office (*n* = 1); several academics from various EU universities and organizations (*n* = 10); early stage researchers and university students (*n* = 5); and national and international NGOs (*n* = 2). A total of ~ 30 experts, overall, eventually attended and engaged in the multi-stakeholder consultation meeting taking the form of three thematic-based focus groups, ensuring a balanced number of participants and type of stakeholders in each focus group (~ 10 participants) (Fig. 1).

### Overview of existing school health policies and programs

Experts discussed the various school health policies and programs. School health programs in EU schools encompass a suite of activities and campaigns. Primary healthcare services represent a key pillar of school health services that are regularly conducted by visiting doctors and school nurses. However, these health screenings, albeit common for countries like Singapore [19] or Japan [20], are not usually implemented on an annual basis for the same children in European countries, as they climb up the ladder of education either in the primary, secondary/vocational or university-level schools; further, it is common that such services are offered to a small student subpopulation sample.

Participants referred to European policies for schools, including, the European Child Guarantee policy [21] that emphasizes on the social needs of children and on “Obtaining effective and free access to quality healthcare.” The first EU HPS network was established in 1992, while the 2015 EU conference on health and education declared that every school shall be an HPS; this initiative is supported by the EU-wide Schools for Health in Europe Network Foundation (SHE) [22] comprising > 45 countries in the European region and some Central Asian countries. The OECD Directorate for Education and Skills collects regularly data on education systems from most countries, including well known programs such as the PISA survey, the International Early Learning and Child Well Being Study, among others [23]. The WHO Regional Office for Europe provides technical assistance to countries for implementing the European framework for quality standards in school health services and competencies for school health professionals [24]. There is also the WHO European Region Child and Adolescent Health and Wellbeing Strategy (2025–2030) that tackles key determinants of child health with emphasis on enhancing the implementation of policies to improve physical and mental health for both children and adolescents [25]. According to the European Parliament’s STOA report on the human exposome, children’s health is one of the “7 C’s” EU policy area initiatives for applications of exposome research [5].

Participants also addressed global initiatives for children’s health in schools like the FRESH partnership, which is a coalition of several UN agencies and global NGOs about promoting basic education, health, safety, equity, including socioeconomic, sustainable and human development across schools, agencies, and systems [26, 27]; the GIZ Fit for School program reinforces the importance of hygiene, physical activity, and proper nutrition in fostering children’s well-being [28]. The WHO HPS global standards aim at benefitting over 2.3 billion school-age children, contributing towards the target of achieving “1 billion lives made healthier” [29].

### Identified themes: challenges and needs in schools

**Fig. 2:**
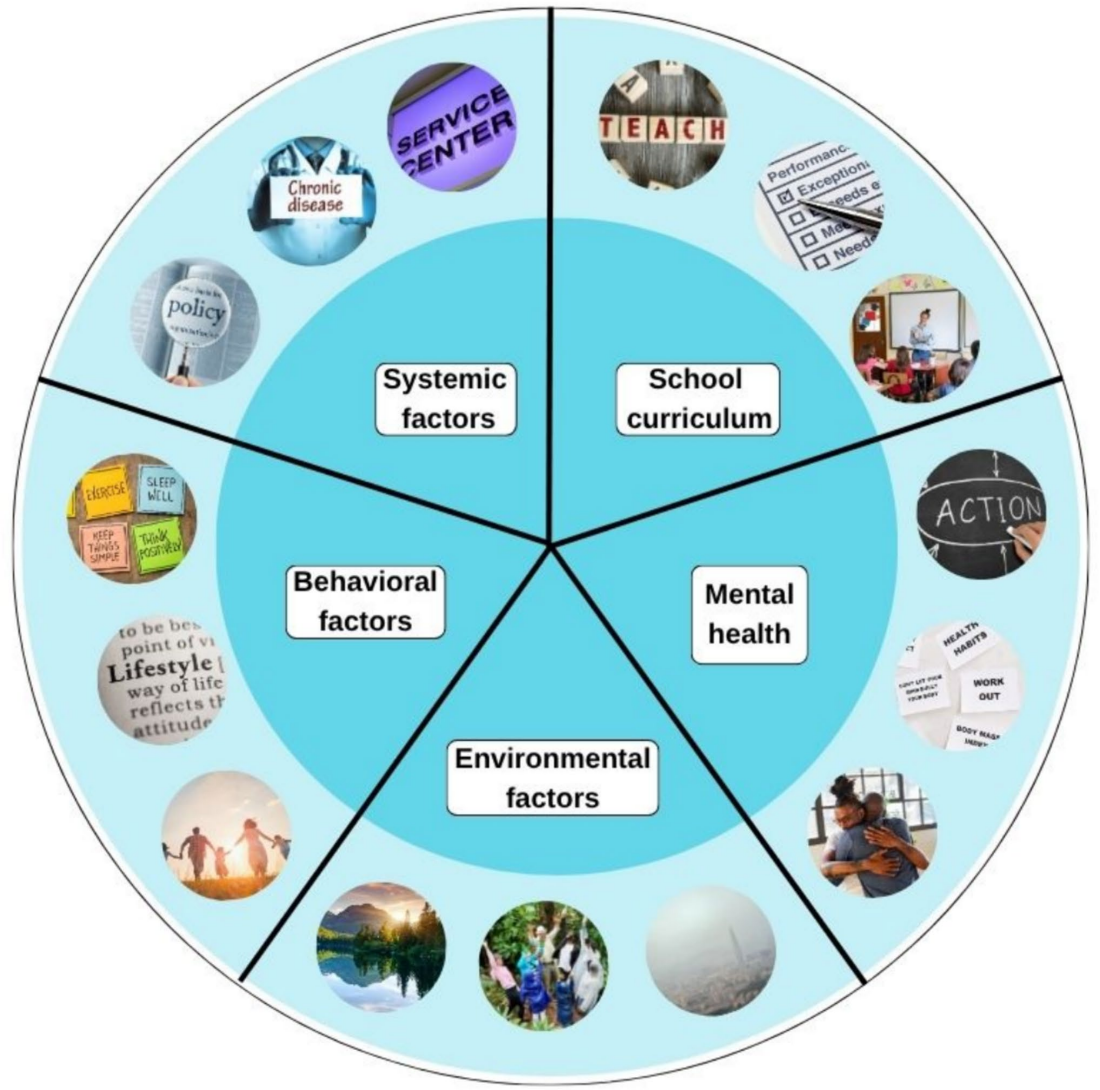
Conceptual framework diagram showing how systemic, curriculum, environmental, behavioral, and mental health factors interact in the school ecosystem

The experts in this multistakeholder consultation consistently highlighted the major challenges and themes that emerged via the focus groups’ thematic analyses below (Fig. 2).

#### Systemic challenges

Participants highlighted that despite the importance and added value of school health initiatives, several programs face systemic challenges that hinder their long-term impact and effectiveness. One of the primary issues is the challenge of long-term planning and sustainable funding, which often results in fragmented, short-lived interventions rather than being comprehensive and enduring programs. This challenge is exacerbated by limited institutional support, preventing health initiatives from becoming fully integrated into the educational system [30]. A key consequence of these gaps is unequal access to health promotion programs, with urban schools and often those geographically located in areas with higher socioeconomic status frequently benefiting from more resources than rural or underserved areas, thereby maintaining children’s health disparities.

Another major obstacle that was highlighted was the insufficient cross-sectoral collaboration between health administration and ministries of education or local governments and local school administrations. The challenge of coordination among these entities and the legal regulations on the capacity of each entity lead to disconnected policies and fragmented health education/promotion interventions, reducing their overall effectiveness and overall societal impact. Additionally, divergent priorities among stakeholders make it difficult to establish a cohesive national strategy for children’s health, as different groups may focus on competing objectives rather than a unified vision. Teachers, who play a crucial role in implementing school health programs, also face significant challenges, including high levels of stress due to heavy workloads, inadequate mental health support, and limited training in health-related initiatives. These factors reduce their capacity to effectively promote students’ well-being [31].

Furthermore, many schools struggle with inadequate awareness and limited compliance with existing regulations, such as those governing school nutrition standards, physical activity requirements, and environmental health policies [32]. The limited policy implementation and/or challenge of enforcement undermines efforts towards healthier and health-enabling school environments. Low community engagement presents another barrier, as parents and local organizations are often uninformed about or insufficiently involved in school health initiatives.

#### Health literacy—curriculum needs

Experts unanimously agreed that a comprehensive health education curriculum is essential for equipping children with lifelong skills to maintain their wellbeing and quality of life. Health literacy is a key competence that all students shall cultivate at school [29]. However, many educational programs in European countries struggle with significant gaps that hinder the systematic implementation of cost-effective health literacy and education programs in the school curriculum. One of the primary challenges is the insufficient integration of health education, particularly in secondary schools, where adolescents may face heightened health risks related to mental health, substance use, and lifestyle-related burden of disease.

Participating experts agreed that the limited classroom time allocated to health promotion/education topics, hinders health education and promotion progress in schools of the post COVID-19 era. This challenge undermines students’ ability to develop sufficient levels of health literacy and to be able to understand, critically appraise and use information related to their health; more than understanding, students shall be able to apply and use health information by forming opinions, judging the quality of information, and making healthy choices and informed decisions [33]. Additionally, overloaded school schedules leave little room for extracurricular health programs or physical activities.

Environmental health education is also notably absent from many school curricula, preventing children from fully appreciating the interconnectedness of health, climate, environment, and planetary sustainability [34]. Similarly, comprehensive sexuality education often receives inadequate teaching time, limiting students’ ability to make informed decisions about their reproductive health, relationships and personal safety [35].

#### School physical environment

Experts agreed that the physical environment of schools plays a crucial role in shaping students’ health, cognitive development, and overall academic performance. However, many schools unintentionally expose children to various environmental hazards, including poor ambient (indoor) air quality, excessive noise, harmful chemicals and inadequate hygiene, and sanitation facilities, all of which have major consequences for students’ well-being and academic performance. Poor air quality within school buildings—often caused by inadequate ventilation, indoor pollutants, and outdoor air pollution—can lead to respiratory issues, fatigue, and deficits in attention and concentration, ultimately impairing academic performance [36–38]. Exposure to harmful chemicals from building materials, cleaning products, or outdated infrastructure materials (lead, asbestos, polyvinyl chloride floors, mold, etc.) poses additional health risks to children and adolescents in schools [39].

Participating experts consistently highlighted that increased heat stress and associated thermal discomfort in school classrooms is another important risk factor for children’s health, often going unnoticed. Research conducted across 61 countries, involving nearly 14 million students, has revealed that cumulative exposure to high temperatures impairs students’ ability to perform complex cognitive tasks [40]. Notably, students from lower socioeconomic backgrounds experience greater cognitive losses compared to their higher-income peers. Climate adaptation technologies—such as air conditioning, advanced ventilation systems, and heat mitigation strategies significantly reduce the negative effects of overheating. In regions where temperatures frequently exceed 28 °C, classrooms without proper cooling and ventilation may severely impact children’s ability to focus and learn [37–40]. Excessive noise levels from traffic, construction, and poor building acoustics further exacerbate these issues, disrupting lessons, increasing stress levels, and reducing students’ ability to retain information. Limited and often unsafe access to green areas particularly in urban settings is another major issue for school communities located in such settings. Limited access to clean water, proper sanitation, and hygiene facilities remain a critical problem, particularly in low- and middle-income countries, or in peri-urban or rural areas of high-income countries without centralized water treatment. Insufficient hygiene infrastructure not only increases the risk of infectious diseases but also disproportionately affects girls, who may struggle to manage menstrual hygiene in inadequate facilities, leading to increased absenteeism. Furthermore, concerns have been raised about potential health risks associated with prolonged exposure to radiation from Wi-Fi and other digital devices [41], underscoring the need for evidence-based assessments and guidelines to ensure safe learning environments in schools [42].

#### Behavioral risk factors

According to the participating experts, one of the most concerning trends is the rise in insufficient physical activity and poor nutrition, both of which contribute to the growing rates of childhood obesity or underweight [43]. This, in turn, increases the risk of developing chronic conditions such as type II diabetes and cardiovascular disease at an early age. Despite widespread awareness of the benefits of physical activity and a balanced and healthy diet, many children continue to engage in sedentary behaviors and consume foods high in energy, fats, free sugars, and salt/sodium [4].

Another emerging risk factor highlighted by the stakeholders was the growing use of e-cigarettes and vaping among adolescents [44]. While traditional smoking rates have declined in many regions, the popularity of vaping has surged, exposing young people to harmful chemicals and nicotine addiction. The long-term health effects of e-cigarettes remain a concern, prompting the need for stricter regulations, increased awareness, and targeted policy interventions to curb their use among school-aged children. With the widespread availability of smartphones, tablets, and computers, children are spending more time on screens for non-educational purposes, often at the expense of physical activity and sleep. The WHO HBSC survey of 280,000 adolescents reported problematic social media use and being at risk of problematic gaming for 11% and 12% of adolescents, respectively [45]. Problematic users in the HBSC survey showed the least favorable mental and social wellbeing profile and the highest level of substance use [46].

### Mental health

Experts highlighted that schools play a fundamental role in shaping children’s mental health, and yet many institutions have limited resources and infrastructure to adequately support students dealing with stress, anxiety, and social-emotional challenges [47]. Some of the most pressing issues are bullying, social exclusion, and peer pressure. These negative experiences can lead to long-term psychological distress, affecting students’ self-esteem, emotional wellbeing, and academic performance. Without proper intervention, children who experience chronic bullying or social isolation may develop anxiety, depression, or other mental health conditions that persist into adulthood.

Another major challenge is the limited access to school-based mental health services. In many countries, there is a shortage of trained school counselors, psychologists, and mental health professionals who can provide students with the support they need. Without these essential services, children struggling with mental health issues may go undiagnosed and untreated, worsening their emotional difficulties over time. The absence of structured psychological support within schools also places additional pressure on teachers, who are often expected to manage students’ emotional needs despite not having specialized training in mental health support [48]. Many educators also would benefit from lifelong learning opportunities to equip themselves with the necessary knowledge and skills to identify warning signs of distress, or to provide appropriate assistance or referrals to professional help.

## Discussion

The multi-stakeholder consultation was instrumental in reaching a consensus among experts about the forward-looking vision of integrating the complex interplay of physical, mental, socio-emotional, and environmental health aspects of children’s everyday life into the HPS strategy [4, 49]. Moving beyond fragmented approaches and those considering one risk factor at a time, the expert consultation consistently highlighted the incorporation of the human exposome concept, and its tools as previously applied to children’s health cohort studies [50–52] into the strategic whole-school HPS approach. The integration of exposomics tools (i.e., surveys, sensors, biomarkers and multi-omics capabilities) [53] into packages of school interventions and into school health policies and programs is essential, recognizing the cumulative impact of all non-genetic risk factors on children’s growth and developmental profiles in schools and beyond [50–52, 54, 55]. Monitoring children’s exposome in and out of schools as they grow and develop would offer a great opportunity to integrate epidemiological surveillance schemes into health school services by considering multiple children’s exposures. Experts stressed that the lack of cost-effective and pragmatic actionable solutions for NCDs and their risk factors in childhood would be addressed by designing and testing school-based actionable interventions towards environmental health, heat stress, vaping, mental well-being, and digital health literacy [56].

**Fig. 3:**
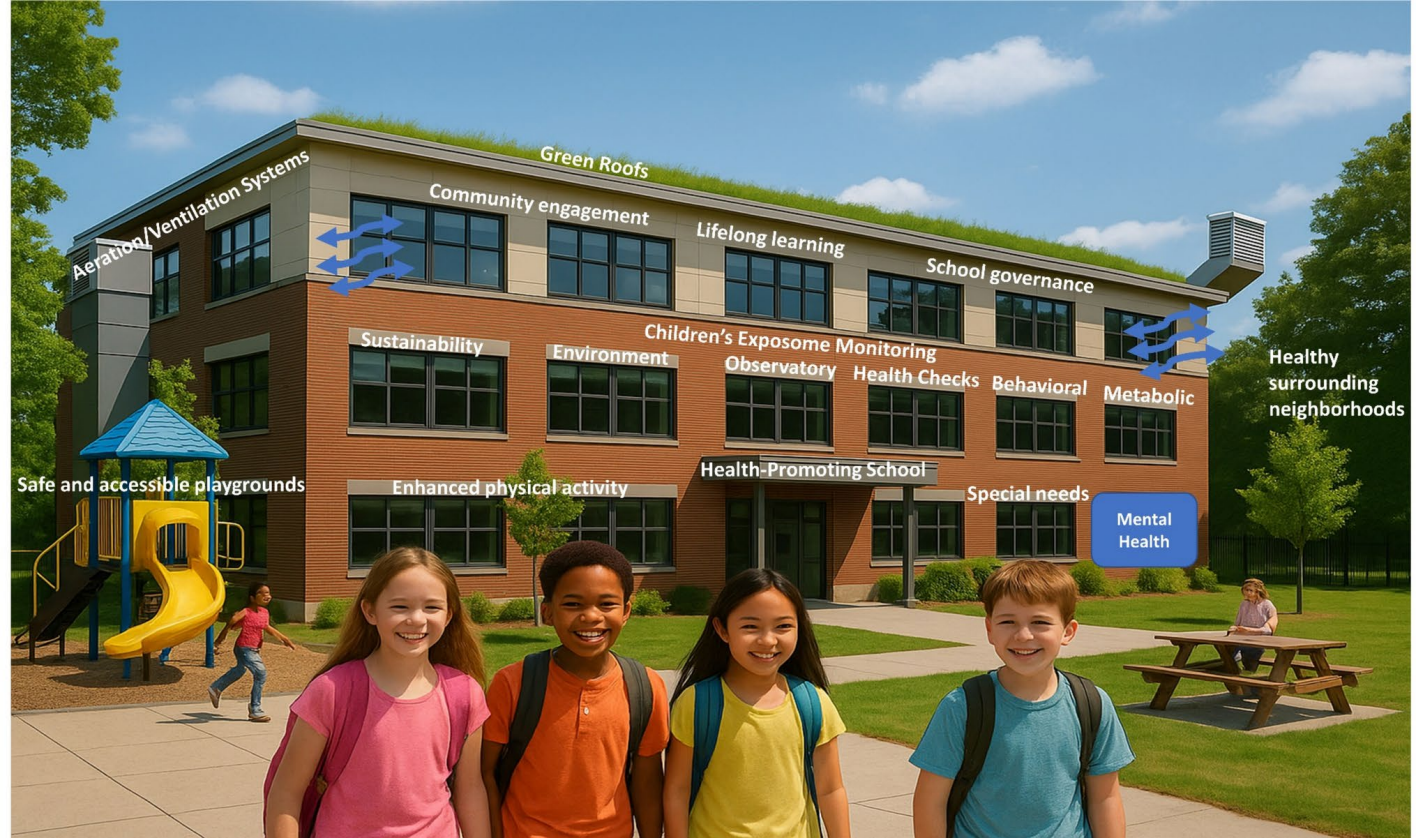
Towards transforming schools to health promoting schools with specific recommendations from the multistakeholder consultation process

The consultation stressed the importance of policies and actions that improve or create school infrastructures, e.g., via systematic health monitoring systems (e.g., observatories [57]) or through better ventilation systems, noise control, through climate-adaptive buildings and classroom designs, or adequate sanitation facilities (Fig. 3). Lifelong training for teachers was clearly emphasized among experts towards equipping them with skills to support a healthy school environment and with the knowledge to systematically engage with the broader school community, fostering healthy behaviors, empathy, and conflict resolution skills.

## Recommendations for the future school health system

The consultation consensus manifested the transformative integration of urban planning, life skills education, translational research, and implementation research in schools. New or improved school infrastructures were suggested to support long-term implementation of the HPS programs, such as school-connected (walking bus) green routes, pedestrian pathways, bike lanes, secure bicycle parking, public open spaces, and road safety measures. Experts recommended a set of empirically chosen interventions (Table 1) calling for an institutionalized system of governance by both the education and health sectors (Fig. 3).

**Table 1:** Workshop-derived set of recommended actions/interventions for European schools towards implementing the HPS strategy using the human exposomics tools

Policy or regulatory actions	• A situation analysis undertaken to assess the current status, assets, needs, and gaps related with the HPS implementation guidance • Legislation needs to reinforce restrictions on electronic and conventional smoking • Regulations to monitor and improve indoor air quality in schools with up-to-date equipment (e.g., sensors), including sound quality indoors and around school premises • Updated school building codes to require improved ventilation systems ensuring better air quality (e.g., installation of air quality improvement technologies) • Continuous Health Education program needs to be implemented across all age groups and classes with no gaps
School Governance	• Creation of joint paths of collaboration on the HPS strategy using experts from both Ministry of Education and Ministry of Health and Social Services together with academics • Incorporation of the needs of parent and teacher Unions into the HPS strategy • The HPS strategy shall also prioritize the development of strategies for childhood diseases, such as obesity, mental health and risk factors such as electronic cigarettes, excessive use of screens for leisure, etc • Strengthening policies mandating the provision of healthy meals and snacks in schools based on the Mediterranean diet, while also better integrating nutrition education in the curriculum • Collaboration between teachers and health professionals by working with children during post-curriculum hours in school, including parental involvement (Iceland model) • Establish regulations ensuring that schools provide access to mental health professionals, such as counsellors and psychologists, to support students’ emotional wellbeing • Encourage parental involvement in mental health initiatives through workshops and resources aimed at educating parents about recognizing and addressing mental health issues in children
Public and environmental health	• Schools should be equipped with appropriate ventilation, toxic-free building materials, and green spaces to reduce harmful exposures and enhance overall wellbeing and school performance • Cool water equipment for free cool tap water should be available in all schools • Surveillance of high air temperature both indoors and outdoors to take preventive measures against overheating classrooms • Ensure only healthy food options are sold in schools • Conduct awareness campaigns that educate communities about the benefits of reduced exposure to environmental stressors and encourage support for school-based interventions • Develop programs that enhance social connections and community support for families, which could improve overall health outcomes for children • Promote healthy dietary habits and physical activity through public health campaigns and education in schools • Increase opportunities for physical activity, such as active recesses, sports programs, and integrating movement into learning. These opportunities can combat sedentary lifestyles
Implementation/Translational research	• Evaluation of ongoing school health programs and policies to provide feedback to policy makers on their effectiveness • Test the effectiveness of interventions promoting healthy childhood development in the context of “no risk society’ and technology use • Support translational research into the longitudinal monitoring of environmental exposures of children in schools and homes together with early-stage markers of chronic diseases, using exposomics tools • Enhance the functionality of environmental and health monitoring observatories to track a wide range of exposures using the human exposome concept and their health impacts that would guide public health interventions and policies effectively • Encourage longitudinal health studies to track the impacts of prenatal and early childhood exposures on long-term health outcomes during critical windows of vulnerability in school aged children • Establish systems for the ongoing monitoring and evaluation of environmental (and other types) interventions in schools to assess their effectiveness on physical, mental and social aspects of health
Education and awareness	• Anti-bullying campaigns and programs promoting inclusivity and peer support for creating a safe and supportive school environment • Educate students about the dangers of (excessive) social media use. Through these apps, online bullying often takes place • Implement mental health education as part of the school curriculum to foster awareness and understanding among students regarding mental health issues • Provide systematic health education topics to children, such as, lessons on air and water quality, noise, waste management, sustainable consumption, etc • Schools should encourage active student-led initiatives to address environmental problems, such as recycling programs, community gardens, or climate action groups • A Health Education training program shall be offered for the rest of the teaching staff, other than health educators • Schools should ensure access to counselors, mental health professionals, and peer support programs to address mental health challenges • Engage parents in health promoting school activities
Urban planning	• Maintaining or increasing accessible natural spaces in urban planning remains crucial due to their known broader health benefits. Safe and easy access to natural spaces is important • Implementing infrastructure improvements that promote safe and well-maintained facilities and spaces for walking, cycling and other physical activity for people of all ages and abilities • Providing enough shade from sun in and around schools (trees but also arcades). Invest in greening school environments through the development of school gardens, green walls, and more accessible natural play areas • Looking into regulations governing sale of tobacco, alcohol and unhealthy food options in areas near schools

A critical component of this transformation is the enhancement of life skills education, equipping students with the necessary competencies to make informed health decisions, manage stress, and develop resilience in an increasingly complex world. Simultaneously, teacher training schemes must be expanded to provide educators with the knowledge and skills required to identify and support students’ physical and mental health needs. Schools ought to become hubs for continuous professional development, where teachers are empowered to integrate health promotion and sustainability strategies into daily interactions with students.

Another essential pillar of progress would be the promotion of translational research opportunities to enable regular evaluation and assessment of school health systems, primary healthcare programs, and health policies. Continuous assessment and adaptation are necessary to ensure that health and sustainability interventions remain relevant, effective, and aligned with evolving public health and social challenges. The implementation of novel frameworks, such as the exposome methodological framework into epidemiological surveillance schemes of primary health care within school communities, would eventually help policymakers and scientists to better understand the cumulative impact of environmental, social, and behavioral/lifestyle exposures on child development, leading to more HPS-centered interventions in schools. Examples could be observatory infrastructures that provide the foundations for long-term monitoring of environment and health inequalities in school communities; the case of the exposome-based CHILDREN_FIRST health and environment observatory in 22 school communities of Cyprus was highlighted [57].

Effective school governance is also paramount, requiring the active involvement of all stakeholders, including policymakers, educators, healthcare professionals, parents, students, and local communities-to co-develop strategies and public health campaigns within each school community (Fig. 3).

## Limitations

The proposed reform perspective exercise suffers from a few limitations. Findings are not transferable beyond the European context, and local sociopolitical dynamics may limit generalizability; findings are intended to be analytically transferable rather than statistically generalizable. It is inevitable that certain topics and views may be subject to selection/desirability biases, while the pool of experts may not be representative of the overall European population. The researcher biography and professional backgrounds would classify as bias-based limitations that are specific to expert consensus exercises in such qualitative research exercises. The nature of the focus groups of this consultation did not allow for ensuring expert participants’ anonymicity.

## Conclusion

This work summarized the expert consensus findings on the challenges, gaps, prerequisites and recommendations to further improve health and educational outcomes for children in schools. The main message of this multi-stakeholder consultation was to apply holistic methodological frameworks, such as that of the human exposome and its exposomics tools towards better facilitating the HPS strategic implementation plan in the school community. This forward-looking vision of systematically observing and intervening where and how children learn and play will help cultivate a healthier, more resilient young generation, capable of thriving in an ever-evolving world.

## Supplementary Information

Below is the link to the electronic supplementary material.

ESM1 (DOCX 36.6 KB)

## Data Availability

No datasets were generated or analysed during the current study.

## Declarations

### Positionality statement

This multistakeholder consultation comprised of professionals that come from diverse yet complementary stakeholder groups specializing in school administration and governance, teaching methods, children’s health and policy making. We acknowledge that all of us carry our own biases on these topics, and we understand that this might introduce some bias due to our personal biographies. We all tried to minimize these by reinforcing the collective integrity of the truth-seeking process in this consultation.
