# Supplementary material for: Reforming schools into health promoting schools: perspective based on expert consensus from a European multistakeholder consultation
Source: Eur J Pediatr. 2026 Feb 9;185(2):126. doi: 10.1007/s00431-025-06736-y (PMC12886229; doi:10.1007/s00431-025-06736-y)
Supplement: Supplementary file 1 — (DOCX 36.6 KB) [file 431_2025_6736_MOESM1_ESM.docx]

# Supplementary Information 1. Guide for the focus group interviews

During the 10^th^ public health workshop of Cyprus (October 08-09, 2024) entitled “Healthy Schools- Healthy Children”, a consultation with stakeholders will take place between multi-stakeholders.

Please be informed that participating experts will be split into 3 main focus groups.

Group 1: Understanding the needs and obstacles of school health promotion

Group 2: Analysis of existing policies and programmes related to health promoting schools

Group 3: Proposal to address priorities, measures and mechanisms for schools.

Each one of you will be part of the three main focus group themes that have been a priori set. The aim is to offer your input and perspectives on the school health issues that the school community faces and what would be the future needs, opportunities and recommendations towards the establishment of a health promoting school.

Please gather your thoughts for the following four questions to be discussed in the event. You will be asked to consider the following technical questions:

Q1 What are the different health and educational needs of children in schools?

Q2 What are the obstacles and needs for a school to become a health promoting one?

Q3 Which are the current school health policies and services operating in Europe?

Q4 What are the main priorities and recommendations for the future health promoting school?
